# Supplementary material for: Indian Ocean Dipole leads to Atlantic Niño
Source: Nat Commun. 2021 Oct 12;12:5952. doi: 10.1038/s41467-021-26223-w (PMC8511204; doi:10.1038/s41467-021-26223-w)
Supplement: Supplementary file 1 — Supplementary Information [file 41467_2021_26223_MOESM1_ESM.pdf]

Supplementary Information for

Indian Ocean Dipole leads to Atlantic Niño

Lei Zhang[1*] and Weiqing Han[1]

1 Department of Atmospheric and Oceanic Sciences, University of Colorado, Boulder, Colorado, USA

*email: lezh8230@colorado.edu

<u>Contents of this file</u>

Supplementary Figures 1-14

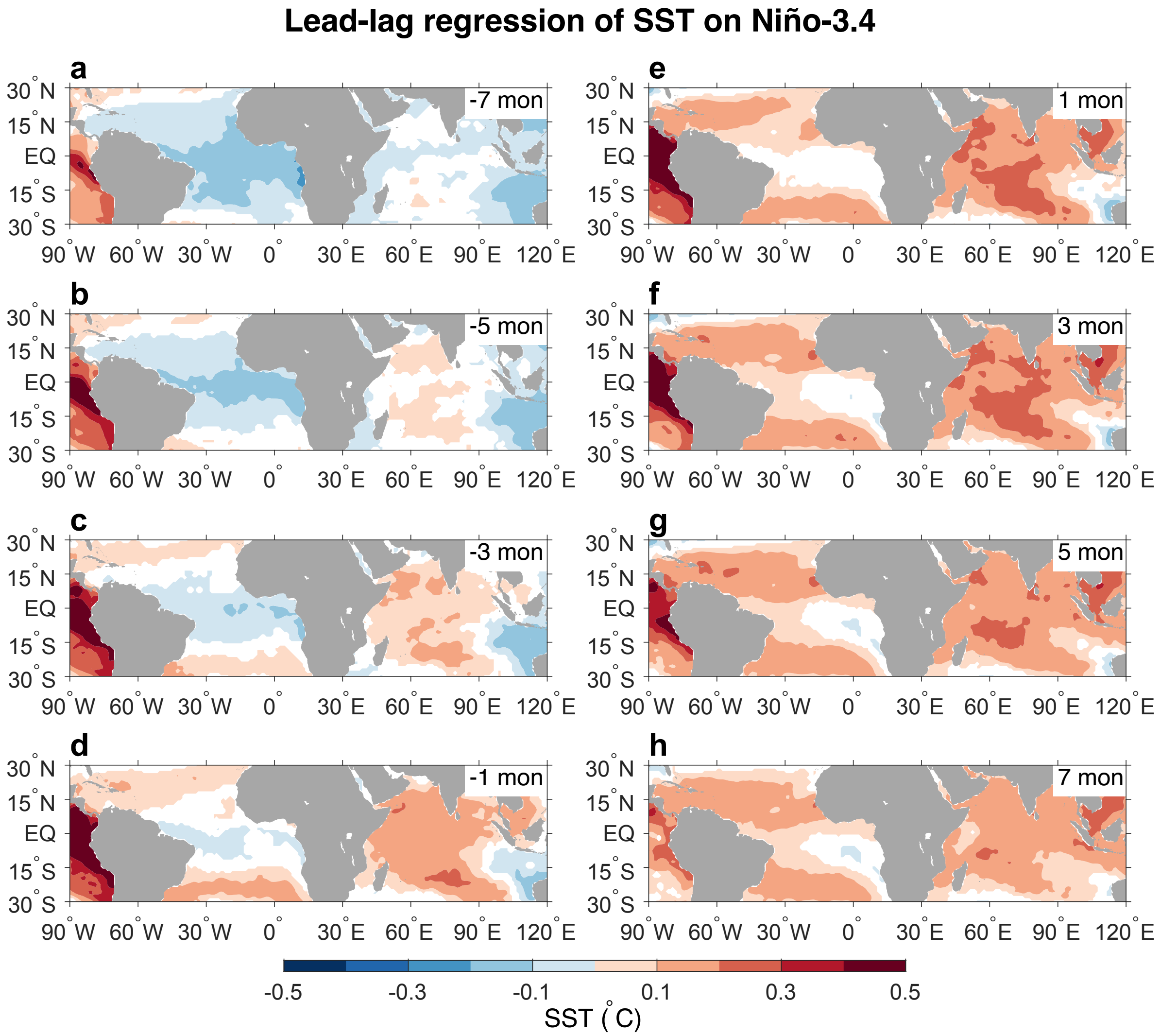

Figure 1 | Impact of ENSO on tropical Atlantic and Indian Ocean sea surface temperature anomalies (SSTA). Lead-lag regression of monthly SST (shading; $^{\circ}C\ ^{\circ}C^{-1}$) and 850 hPa wind anomalies (vector; $m\ s^{-1}\ ^{\circ}C^{-1}$) on the monthly Niño-3.4 index. **a-h** Results for –7 to 7 months leading time, with negative representing prior to ENSO peak and positive for after ENSO peak. Shading represents anomalies that are statistically significant at the 90% confidence level.

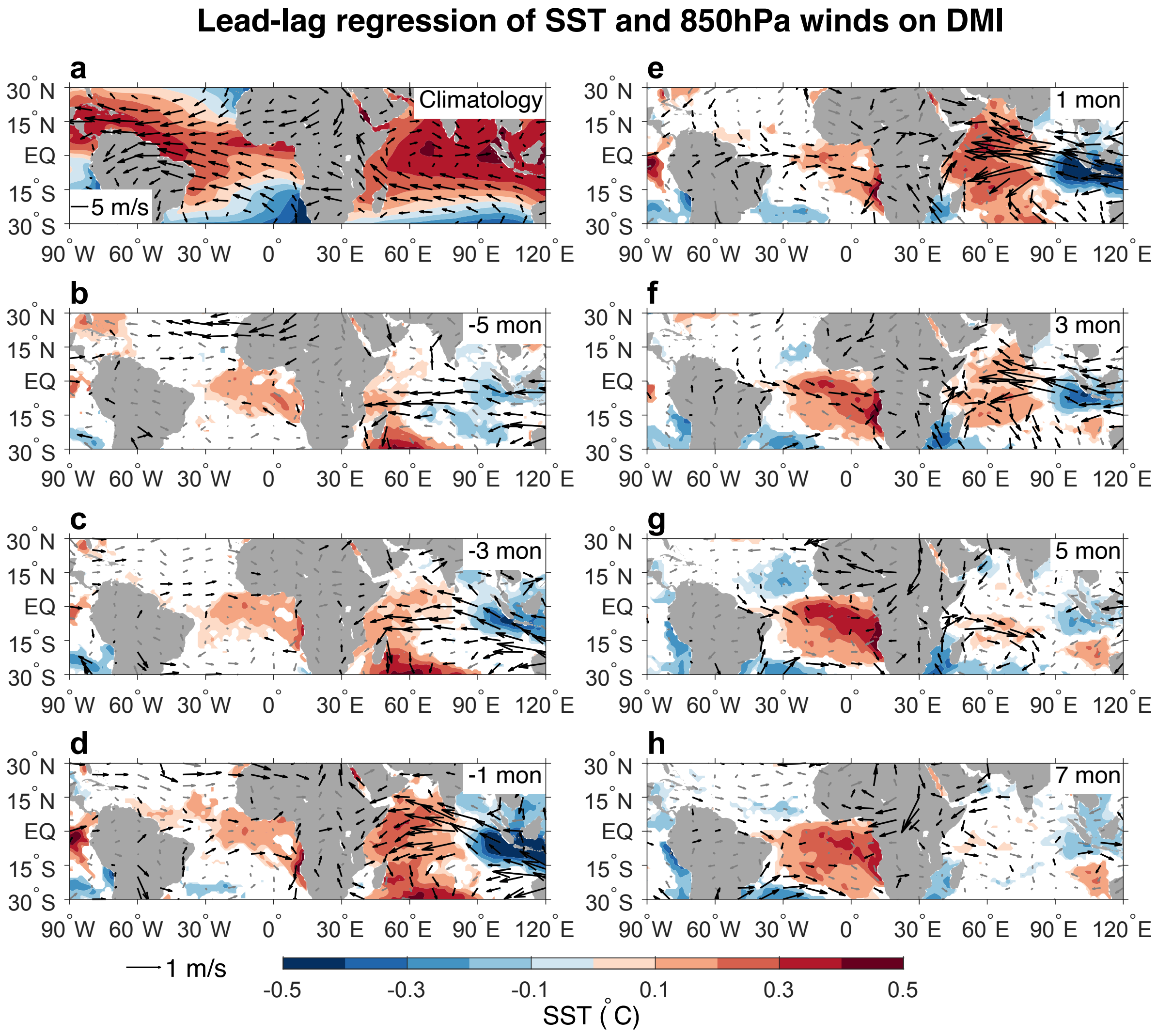

Figure 2 | Impact of positive IOD on Atlantic Niño with ENSO influence removed. a Annual mean climatology of sea surface temperature (SST) (˚C) and 850hPa wind (m s^{-1}). **b-h** Lead-lag regression of monthly SST anomalies (SSTA; shading; ˚C ˚C^{-1}) from the Hadley Centre Sea Ice and SST (HadISST) and 850 hPa wind anomalies (vector; m s^{-1} ˚C^{-1}) from the European Centre for Medium-Range Weather Forecasts (ECMWF) twentieth century reanalysis (ERA-20C) on monthly Dipole Mode Index (DMI), which represents the Indian Ocean Dipole (IOD) mode. ENSO influences have been removed prior to the analysis by removing the 3-month lead-lag regression on the Niño-3.4 index. **b-h** Results for –5 to 7 months leading time, with negative representing prior to IOD peak and positive after IOD peak. Shading and black vectors represent anomalies that are statistically significant at the 90% confidence level.

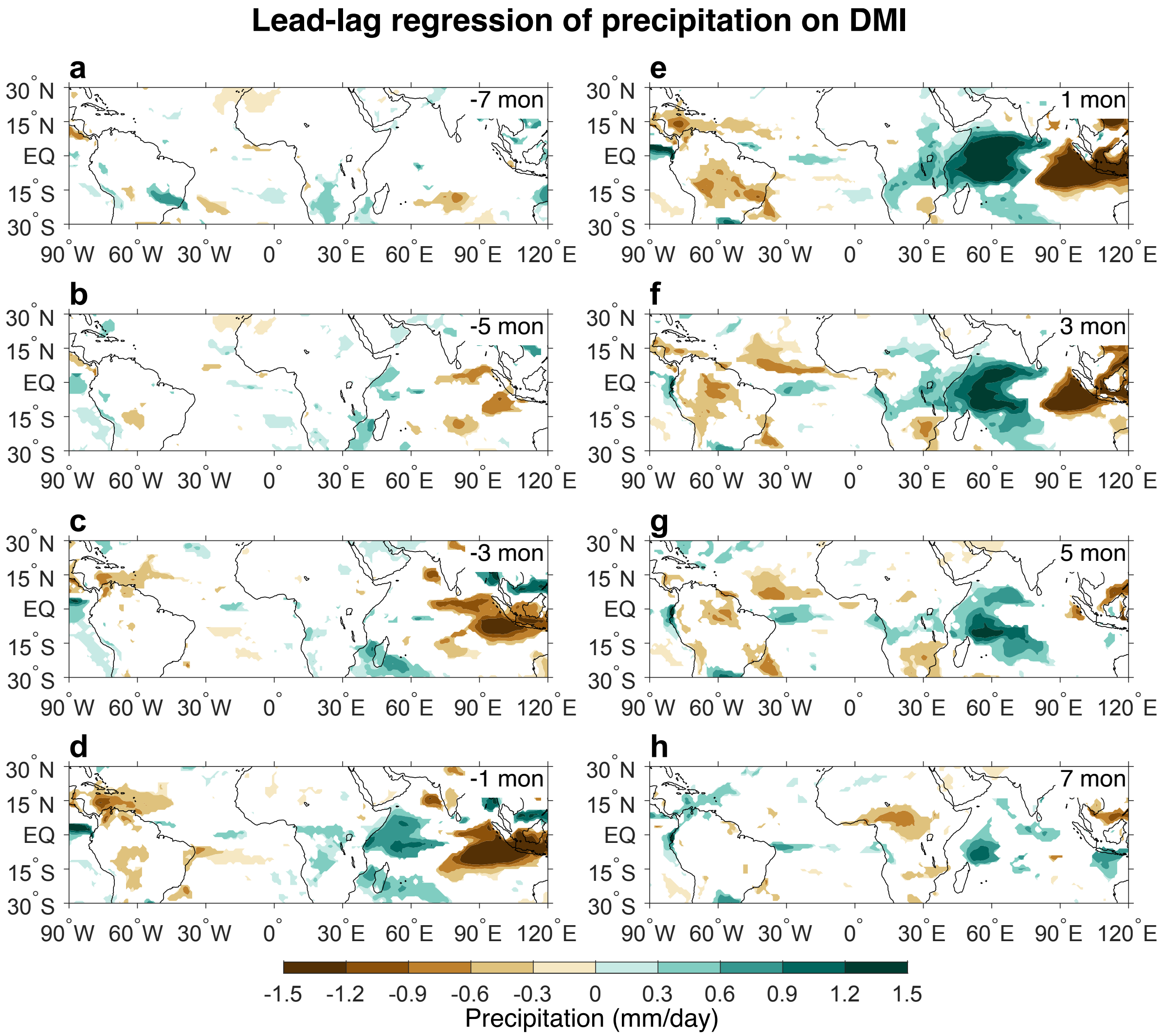

Figure 3 | Precipitation anomalies associated with the positive IOD. Lead-lag regression of monthly precipitation (shading; mm day^{-1} °C^{-1}) on the monthly Dipole Mode Index (DMI). **a-h** Results for –7 to 7 months leading time, with negative representing prior to Indian Ocean Dipole (IOD) peak and positive after IOD peak. Shading represents anomalies that are statistically significant at the 90% confidence level.

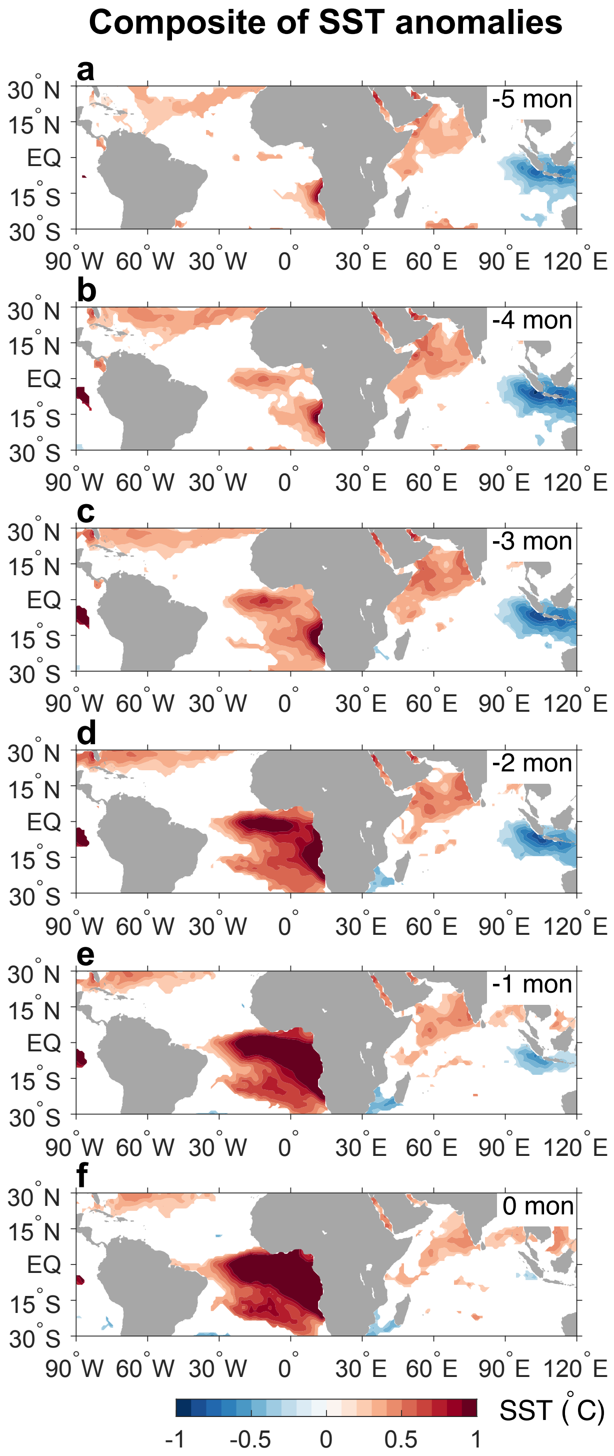

Figure 4 | Tropical Atlantic sea surface temperature anomalies (SSTA) associated with the positive IOD. Composite differences of 3-month running mean SSTA between selected Atlantic Niño and Niña events that are associated with the positive and negative IOD forcing (see Methods section), respectively. Unit is ˚C. **a-f** Results from month –5 to month 0, with month 0 representing the peak of the Atlantic Niño. Negative means months prior to the Atlantic Niño peak. Shown are results that are statistically significant at the 90% confidence level.

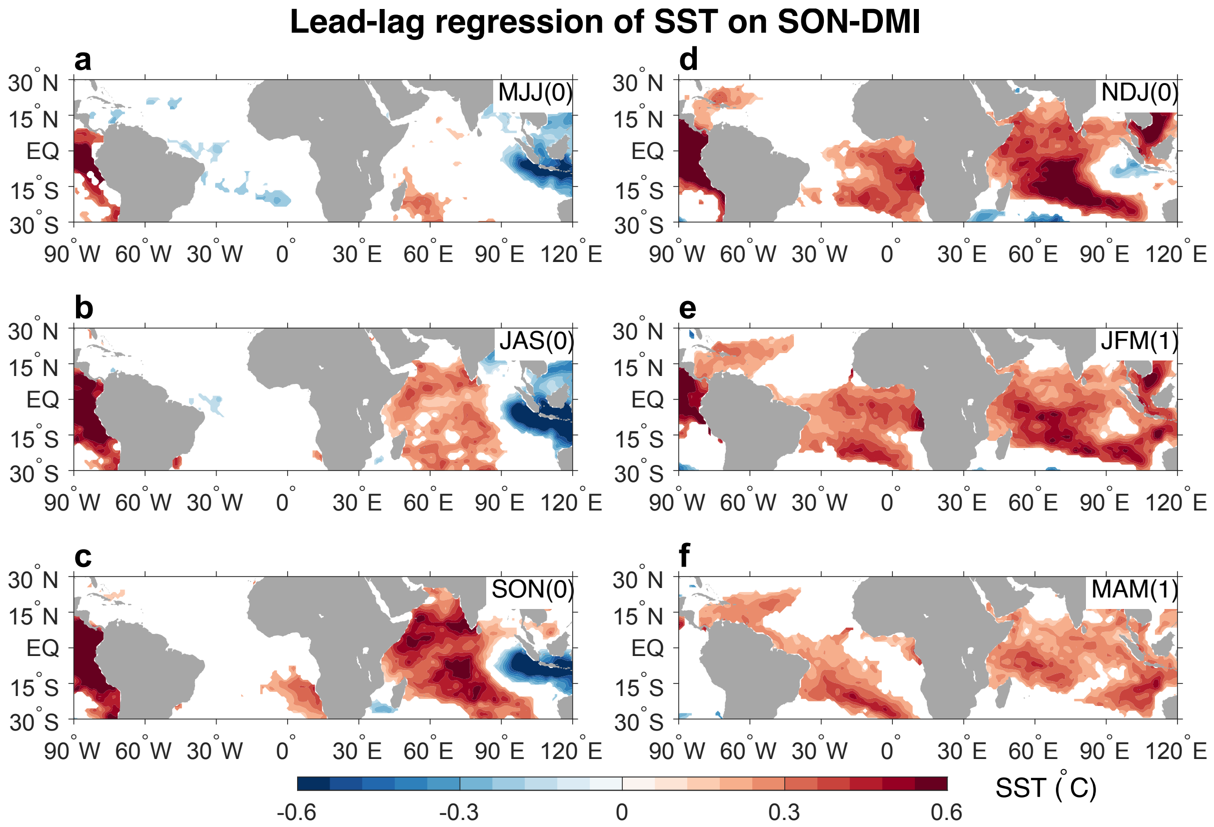

Figure 5 | Seasonal mean tropical Atlantic sea surface anomalies associated with the positive IOD. Lead-lag regression of 3-month mean sea surface temperature anomalies (SSTA) on September-November (SON)-mean DMI. Units are ˚C ˚C^{-1}. **a-f** Results for –4 to 6 months leading time, with negative represent prior to the Indian Ocean Dipole (IOD) peak in SON. Shown are results that are statistically significant at the 90% confidence level.

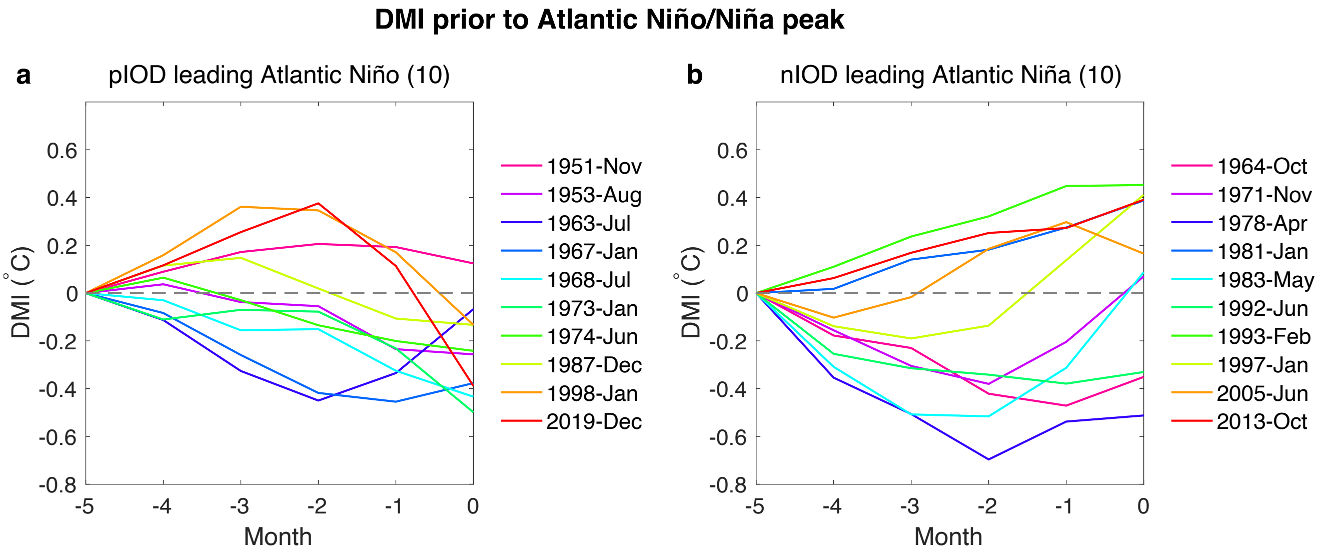

Figure 6 | Evolution of DMI prior to the Atlantic Niño/Niña peak. Evolution of 3-month running mean Dipole Mode Index (DMI) during selected **a** positive Indian Ocean Dipole (pIOD)-associated Atlantic Niño and **b** negative Indian Ocean Dipole (nIOD)-associated Atlantic Niña events (see Methods section). Shown are DMI from month –5 to month 0, with month 0 representing the peak of Atlantic Niño/Niña. The value of DMI in month -5 has been subtracted for each event.

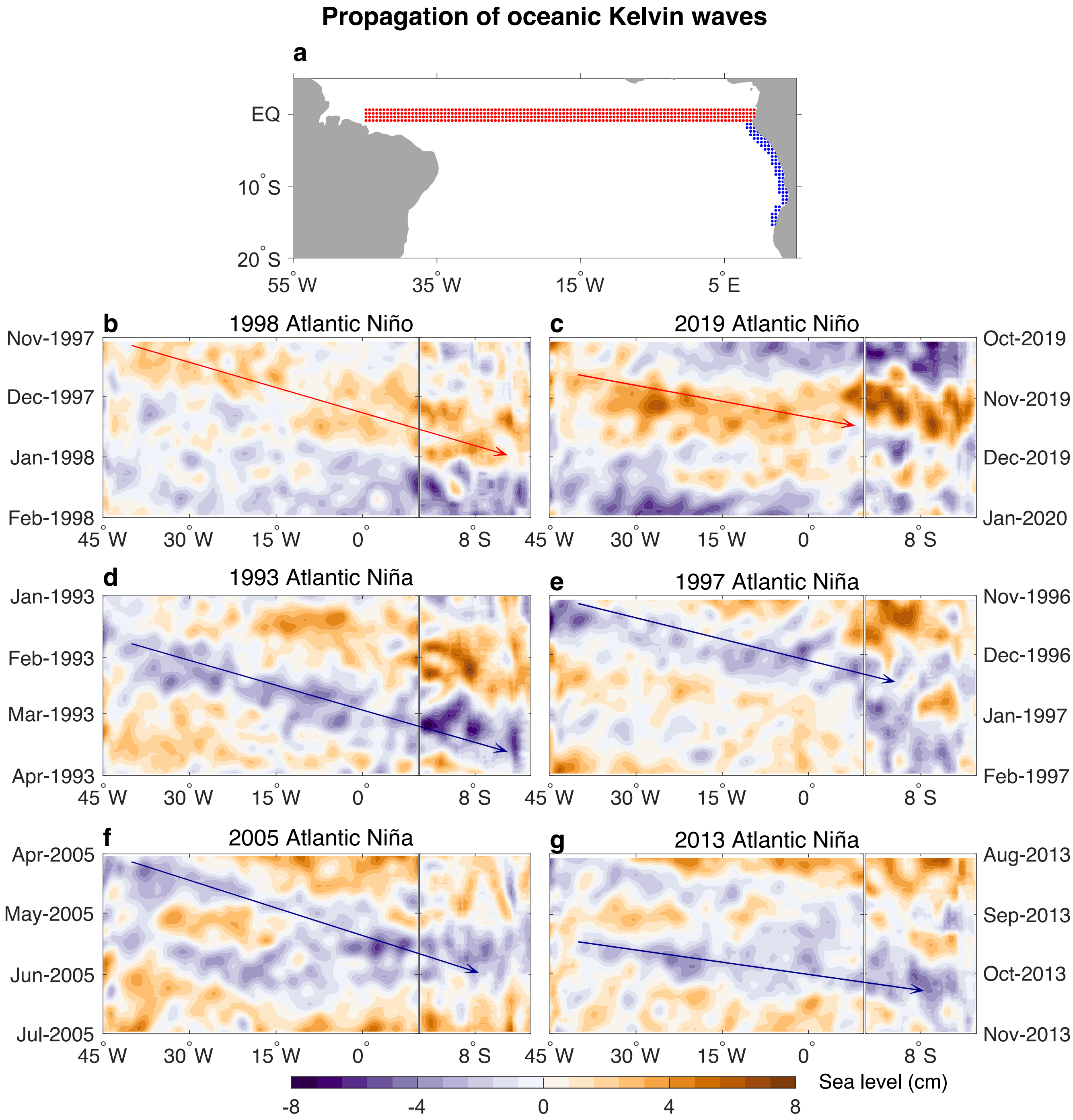

Figure 7 | Oceanic Kelvin waves during Atlantic Niño/Niña. a Regions used to calculate the sea level anomalies in **b-g**. **b-g** Hovmöller diagrams showing sea level anomalies along the Atlantic equator and along eastern boundary, the red and blue regions shown in **a**. Unit is cm. The seasonal mean value at each grid point has been removed. Red and blue arrows mark the propagation of equatorial and coastal Kelvin waves during Atlantic Niño and Niña, respectively.

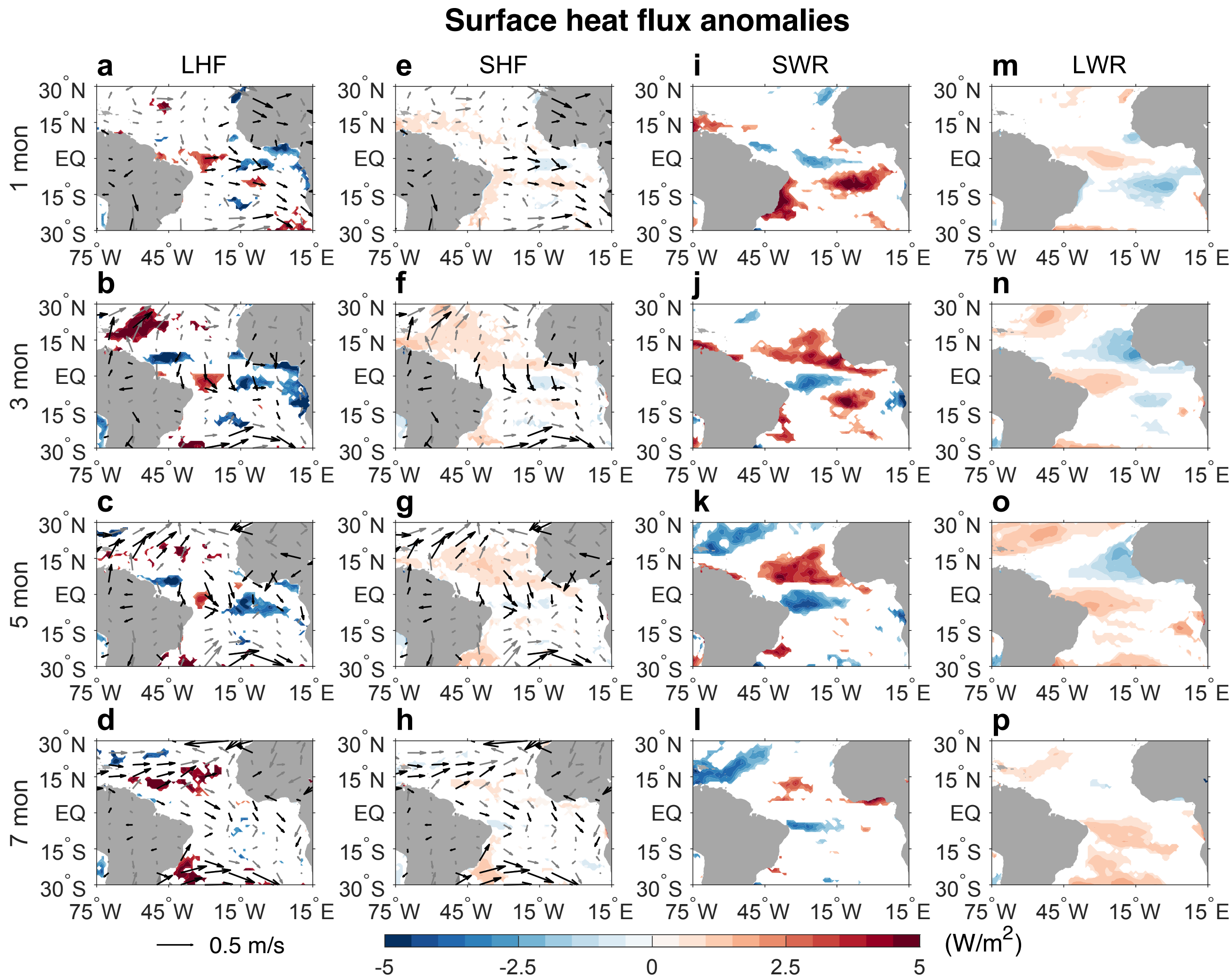

Figure 8 | Surface heat flux anomalies during Atlantic Niño. Lead-lag regression of surface heat flux anomalies on the Dipole Mode Index (DMI). Units are W m^{-2} $^{\circ}C^{-1}$. Shown are regression of **a-d** surface latent heat flux (LHF), **e-h** surface sensible heat flux (SHF), **i-l** surface shortwave radiation (SWR), and **m-p** surface longwave radiation (LWR). Positive means ocean receives heat. Vectors denote regression of the surface wind anomalies (m s^{-1}). Shown are results after the Indian Ocean Dipole (IOD) peak. Shading and black vectors represent anomalies that are statistically significant at the 90% confidence level.

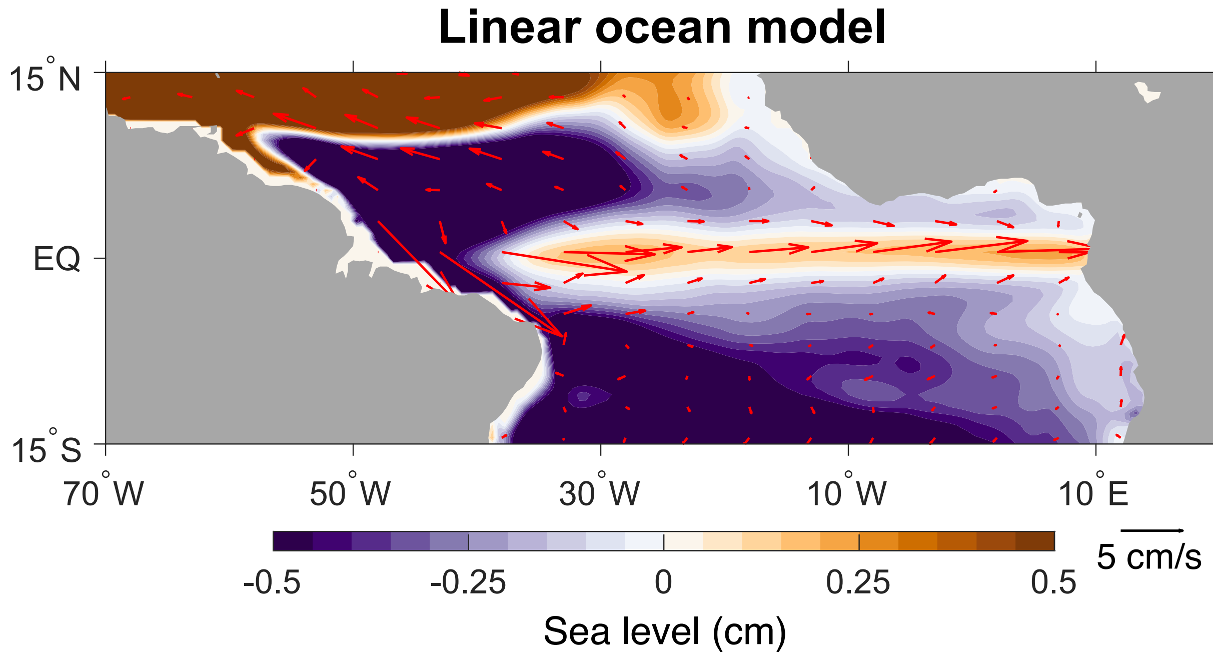

Figure 9 | Linear ocean model results. Sea level (shading; cm) and ocean current anomalies (vector; cm s^{-1}) in a linear ocean model. The ocean model was forced by differences of June-November surface wind stress anomalies between the two sets of atmospheric general circulation model (AGCM) experiments that are forced with sea surface temperature anomalies (SSTA) of positive and negative Indian Ocean Dipole (IOD), respectively (see Fig. 4). Shown are the results averaged between June and November.

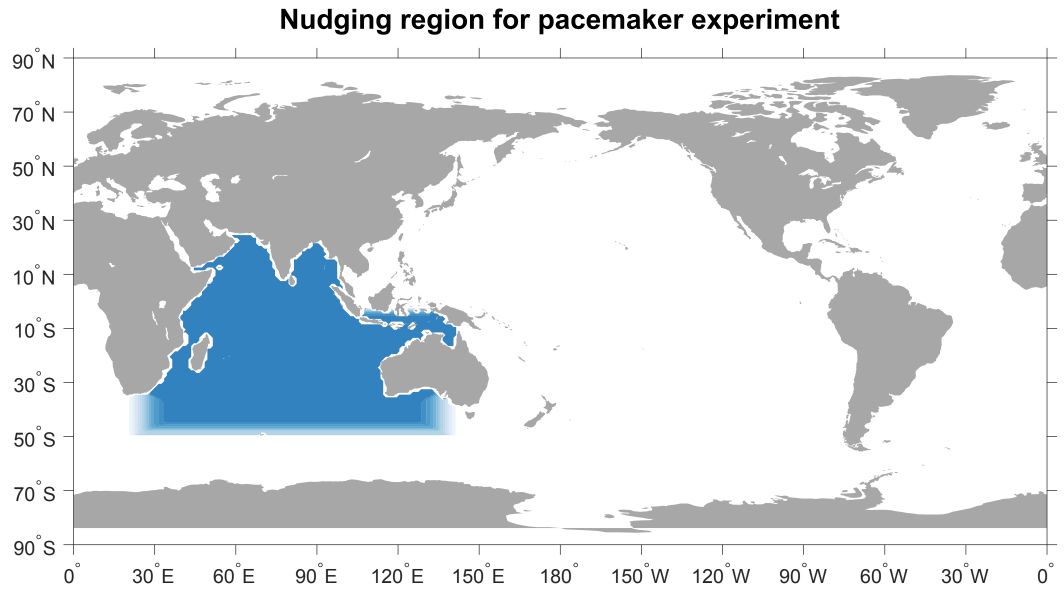

Figure 10 | Nudging region for the Indian Ocean pacemaker experiment. Blue shading represents the nudging region in the Indian Ocean pacemaker experiment where the sea surface temperature anomalies (SSTA) are restored to observed values. A sponge layer is added at the western, southern and eastern boundaries.

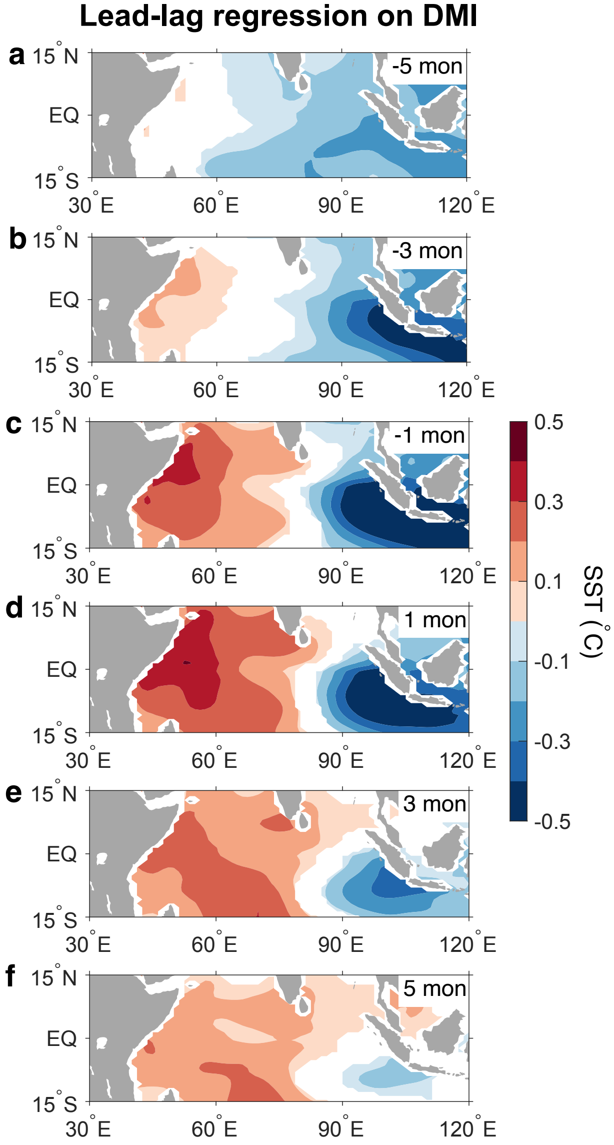

Figure 11 | Evolution of positive IOD in the Indian Ocean pacemaker experiment. Lead-lag regression of sea surface temperature anomalies (SSTA; shading; ˚C ˚C^{-1}) on monthly Dipole Mode Index (DMI). Shown are ensemble mean Indian Ocean pacemaker experiment results that are statistically significant at the 90% confidence level. **a-f** Results for –5 to 5 months leading time, with negative representing months prior to Indian Ocean Dipole (IOD) peak and positive for after IOD peak.

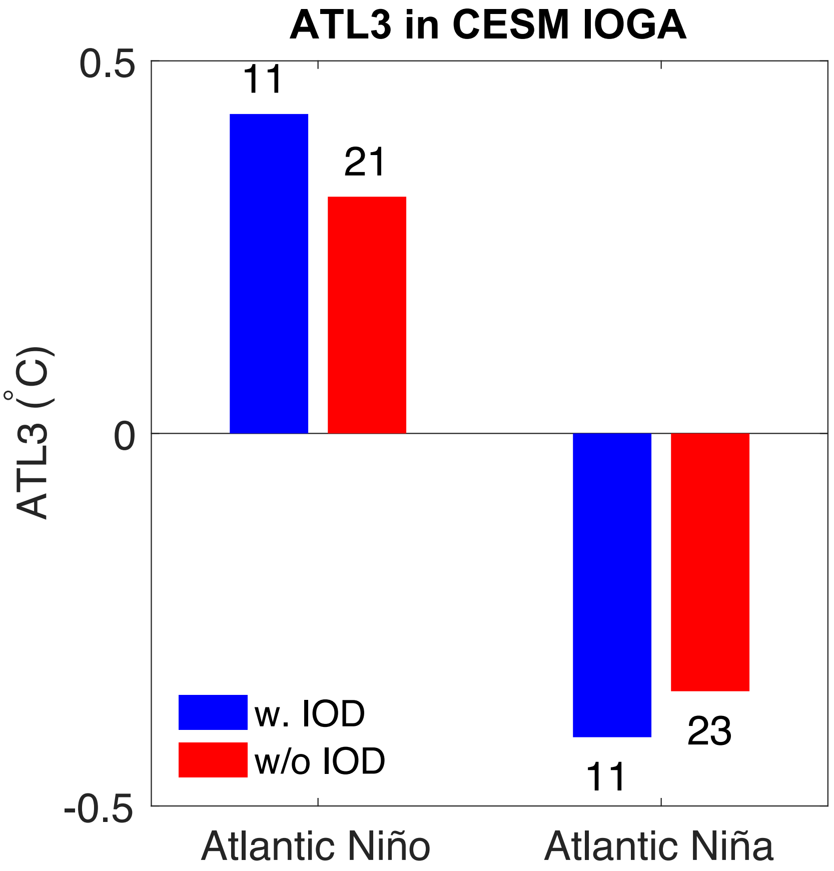

Figure 12 | Amplitudes of Atlantic Niño/Niña events in the Indian Ocean pacemaker experiment. Peak of 3-month running mean ATL3 indices during Atlantic Niño and Niña years in ensemble mean of Indian Ocean pacemaker experiments. Unit is ˚C. Blue bars represent Atlantic Niño or Niña that are associated with the Indian Ocean Dipole (IOD) forcing, and red bars are for Atlantic Niño or Niña events that are independent of the IOD (see Methods section). Numbers of the events for each category are shown at the top or bottom of the bars.

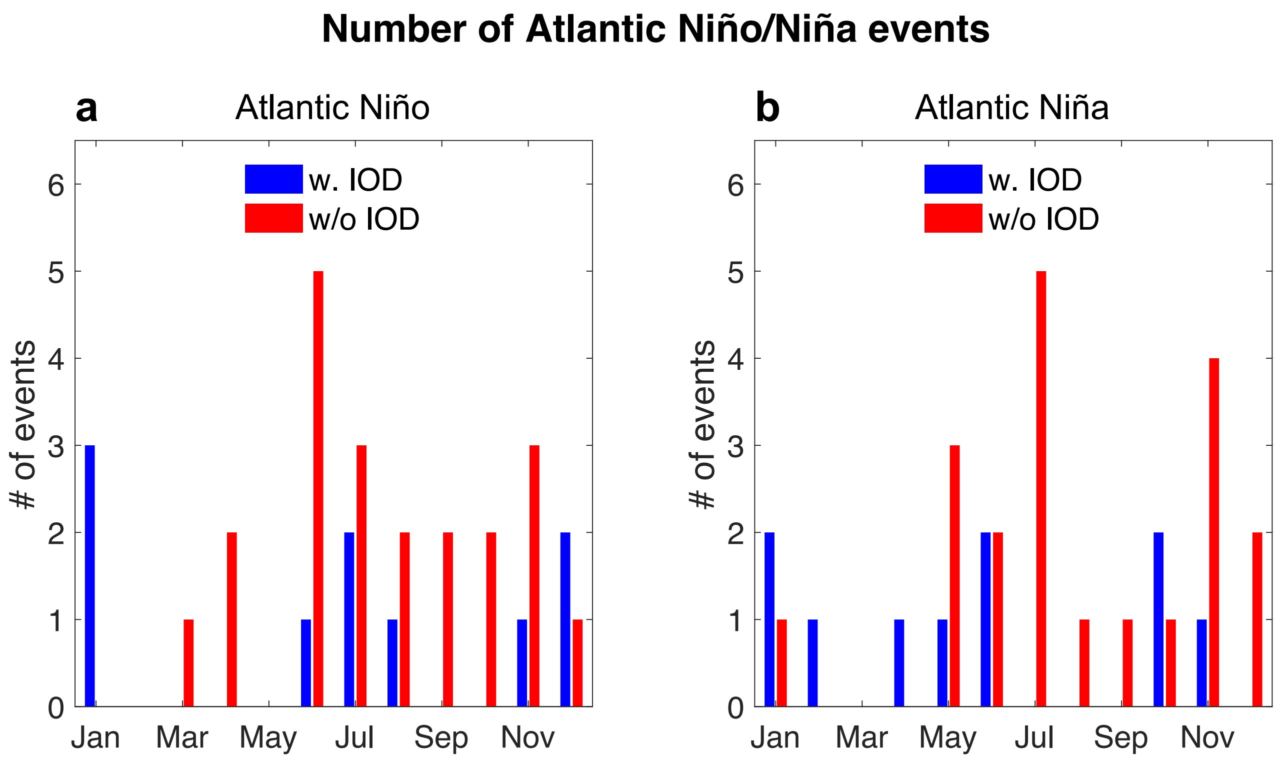

Figure 13 | Number of Atlantic Niño/Niña events in observations. Number of **a** Atlantic Niño or **b** Niña events in each month that are associated with the Indian Ocean Dipole (IOD) (blue) and are independent of the IOD (red) in observations (see Supplementary Fig. 6).

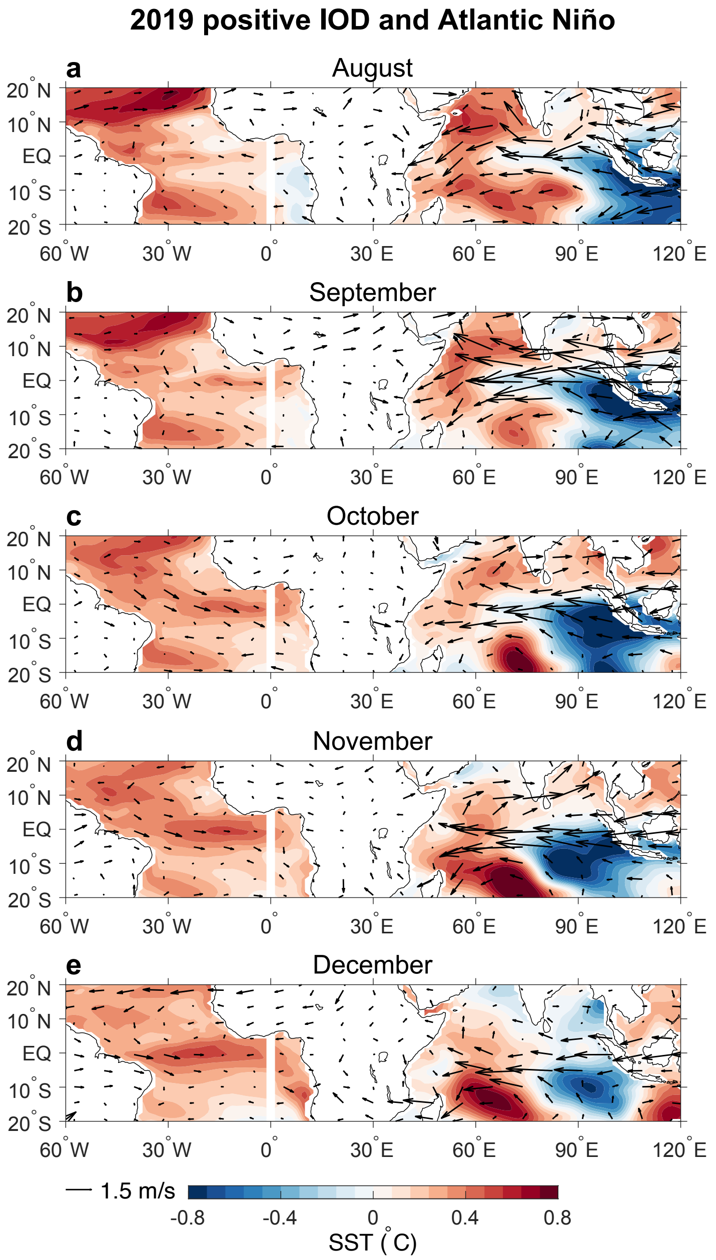

Figure 14 | The positive IOD and Atlantic Niño in 2019. Sea surface temperature anomalies (SSTA; shading; ˚C) and 850hPa wind anomalies (vector: m s^{-1}) in 2019 in the ensemble mean of Indian Ocean pacemaker experiment. **a-e** Results from August to December 2019.
